# Maturation Along White Matter Tracts in Human Brain Using a Diffusion Tensor Surface Model Tract-Specific Analysis

**DOI:** 10.3389/fnana.2016.00009

**Published:** 2016-02-16

**Authors:** Zhang Chen, Hui Zhang, Paul A. Yushkevich, Min Liu, Christian Beaulieu

**Affiliations:** ^1^Department of Biomedical Engineering, Faculty of Medicine and Dentistry, University of AlbertaEdmonton, AB, Canada; ^2^Department of Computer Science and Centre for Medical Image Computing, University College LondonLondon, UK; ^3^Penn Image Computing and Science Laboratory, Department of Radiology, University of PennsylvaniaPhiladelphia, PA, USA

**Keywords:** neurodevelopment, diffusion tensor imaging, fractional anisotropy, mean diffusivity, tractography

## Abstract

Previous diffusion tensor imaging tractography studies have demonstrated exponential patterns of developmental changes for diffusion parameters such as fractional anisotropy (FA) and mean diffusivity (MD) averaged over all voxels in major white matter (WM) tracts of the human brain. However, this assumes that the entire tract is changing in unison, which may not be the case. In this study, a surface model based tract-specific analysis was applied to a cross-sectional cohort of 178 healthy subjects (83 males/95 females) aged from 6 to 30 years to spatially characterize the age-related changes of FA and MD along the trajectory of seven major WM tracts – corpus callosum (CC) and six bilateral tracts. There were unique patterns of regions that showed different exponential and linear rates of increasing FA or decreasing MD and age at which FA or MD levels off along each tract. Faster change rate of FA was observed in genu of CC and frontal-parietal part of superior longitudinal fasciculus (SLF). Inferior corticospinal tract (CST), posterior regions of association tracts such as inferior longitudinal fasciculus, inferior frontal occipital fasciculus and uncinate fasciculus also displayed earlier changing patterns for FA. MD decreases with age also exhibited this posterior-to-anterior WM maturation pattern for most tracts in females. Both males and females displayed similar FA/MD patterns of change with age along most large tracts; however, males had overall reached the FA maxima or MD minima later compared with females in most tracts with the greater differences occurring in the CST and frontal-parietal part of SLF for MD. Therefore, brain WM development has spatially varying trajectories along tracts that depend on sex and the tract.

## Introduction

Quantitative magnetic resonance imaging (MRI) has demonstrated significant brain changes during development from infancy to adulthood *in vivo*. Structural MRI studies have reported age-related changes in brain volumes (Giedd et al., 1999; Good et al., 2001), areas (Thompson et al., 2000), cortical thickness (Sowell et al., 2004; Shaw et al., 2008), and regional gray matter (GM) and white matter (WM) density (Paus et al., 1999; Gogtay et al., 2004). It has been suggested that those developmental changes in GM and WM observed at the macroscopic MRI level may reflect synaptic pruning and myelination at the microscopic neuronal level (Gogtay et al., 2004).

Diffusion tensor imaging (DTI) techniques have enabled the virtual dissection of WM tracts *in vivo* and permit the investigation of tissue microstructure, including indirect measures of myelination and axonal density, that may be more sensitive than conventional imaging to changes with age (Basser et al., 1994; Beaulieu, 2002; Le Bihan, 2003). Using region-of-interest (ROI) by manual placement/tractography or voxel based analysis, brain development studies of children and adolescents using DTI have consistently demonstrated age-related increases of fractional anisotropy (FA) and decreases of overall diffusion mean diffusivity (MD; Klingberg et al., 1999; Morriss et al., 1999; Schmithorst et al., 2002; Schneider et al., 2004; Barnea-Goraly et al., 2005; Snook et al., 2005; Ashtari et al., 2007; Bonekamp et al., 2007; Eluvathingal et al., 2007; Lebel et al., 2008; Tamnes et al., 2010) that differ between tracts with some data suggesting a posterior-to-anterior developmental trend (Colby et al., 2011). The appropriate fit of FA or MD with age depends somewhat on the age span covered. The general course of FA and MD across the lifespan in WM shows average FA values rising throughout childhood, adolescence, peaking between 20 and 42 years of age and then declining, due to aging processes (Han et al., 2007; Lebel et al., 2012). This FA trajectory is mirrored by a similarly decreasing then increasing trajectory with age for MD. However, in neurodevelopmental studies with a limited age range from childhood to adulthood (typically up to 30 years), FA or MD versus age curves have been fit with either an exponential trajectory to account for FA/MD changing faster at younger ages and then leveling off at a plateau (Lebel et al., 2008; Colby et al., 2011; Taki et al., 2013b) or linear model that assumes the same rate of diffusion parameter changes across the given age span (Schmithorst et al., 2002; Barnea-Goraly et al., 2005; Bonekamp et al., 2007).

However, the ROI-based approach requires averaging the scalar values such as FA or MD from all vertices within the ROI, either manually placed on a 2D slice(s) or using tractography in 3D, into a single value for each tract. On the other hand, voxel-based approaches that can assess various portions of tracts simultaneously face issues of inter-subject alignment quality (Jones et al., 2005a; Van Hecke et al., 2009), although methods have been proposed to mitigate these issues, e.g., tract-based spatial statistics, TBSS (Smith et al., 2006), but not without their own limitations (Bach et al., 2014).

The main issue with a global estimate of diffusion parameters over an entire tract is it assumes that the entire length of a tract behaves the same with age and it cannot detect focal changes or regional variations within the tract. The ‘whole brain’ voxel-based approaches that do attempt to pull out regional differences do not address specific tracts *a priori*. This has led to a growing interest in the development of tract specific methods that can spatially characterize quantitative imaging parameters along the trajectory of tracts. The main obstacle in any along-tract analysis is the non-uniform spatial sampling of the vertices within each streamline. To overcome the issue, several approaches have been proposed, such as a scale-invariant parameterization by arc-angle (Gong et al., 2005), fiber clustering and measurement (Maddah et al., 2008; Prasad et al., 2014) and fiber tract parameterization by arc length (Jones et al., 2005b; Corouge et al., 2006; Goodlett et al., 2009; O’Donnell et al., 2009; Zhu et al., 2011; Yeatman et al., 2012). Using those along–tract methods, studies have found WM abnormalities (FA, MD) along several major tracts in brain disorders such as epilepsy (Concha et al., 2012), Alzheimer’s disease (Nir et al., 2015) and fetal alcohol spectrum disorder (Colby et al., 2012). Brain development cross-sectional studies in infants (1–2 years), toddlers (2–4 years), and children/adolescence (9–16 years) using along-tract analysis showed unique changes with age of DTI parameters such as FA along several tracts (Goodlett et al., 2009; Zhu et al., 2011; Yeatman et al., 2012; Johnson et al., 2013). A recent study also found various diffusion parameters that were variable along multiple WM tracts related to sex, hemisphere, and age within a sample of 26–46 months-old typically developing young children (Johnson et al., 2013). The variations in diffusion at different tract locations likely reflect unique rates of axonal myelination and packing (Beaulieu, 2002; Song et al., 2002, 2005) that might be due to local differences in vasculature, supporting glial structure, and biochemistry throughout the brain (Ito et al., 2005; Yeh et al., 2009; Vasung et al., 2010).

However, those methods are best suited for tracts with tubular geometry such as the cingulum as DTI measurements are averaged across the cross-section along the fibers; larger sheet-like structures such as corpus callosum (CC) have to be divided into several tubular bundles. To overcome these limitations, a recent study has suggested a tract-specific analysis (TSA) using a continuous medial representation (cm-rep) to model individual WM tracts (Yushkevich et al., 2006, 2008; Zhang et al., 2010). The advantage of using cm-rep is that the skeleton of the tract is represented by a parametric surface that allows manifold-based statistical analyses similar to those used in cortical surface mapping (Lerch et al., 2006). As it is an atlas-based approach, it can reduce user bias and improve efficiency of analysis for large data sets examining numerous tracts, albeit with the primary limitation of requiring adequate spatial normalization between individuals. This tract surface methodology has not yet been applied to the study of neurodevelopment.

The purpose of this study is to spatially characterize DTI parameter changes using linear and exponential fits along several major tracts using TSA in both males and females in a large cross-sectional cohort of 178 participants from young childhood to adulthood (6–30 years). We hypothesize that each tract will show a unique pattern of diffusion changes that reflects a more localized developmental pattern, rather than having uniform changes along the entire tract, and will have similar patterns between males and females but with different maturation rates and timing.

## Materials and Methods

### Subjects

This study includes 178 healthy right-handed participants (male/female: 83/95) aged from 6 to 30 years from our previous DTI development study that averaged all the FA or MD values across the entire tract (Lebel et al., 2008), although excluding participants who were left-handed or had images that failed the new pre-processing steps (detailed below). Participants were asked a series of questions to ensure there was no history of neurological or psychiatric disease or brain injury. All participants gave informed consent; child assent and parent/guardian consent was obtained for volunteers under 18 years. The age range was similar for males (6–30 years) and females (6–30 years) with similar mean and standard deviations of 15.6 ± 6.1 and 16.0 ± 6.4 years, respectively. This study was approved by the University of Alberta Human Research Ethics Board.

### Image Acquisition

All data was acquired on a single 1.5T Siemens Sonata MRI scanner. Using a protocol set up over a decade ago to enable studies in young children, standard DTI was acquired using a dual spin-echo, single shot echo-planar imaging sequence with the following parameters: 40 3-mm-thick slices with no inter-slice gap, TR = 6400 ms, TE = 88 ms, six non-collinear diffusion sensitizing gradient directions with *b* = 1000 s/mm^2^, eight averages, field-of-view 220 mm × 220 mm, matrix of 96 × 128 zero-filled to 256 × 256, and scan time of 6:06 min.

### Tract-Specific Diffusion Measurements

**FIGURE 1:**
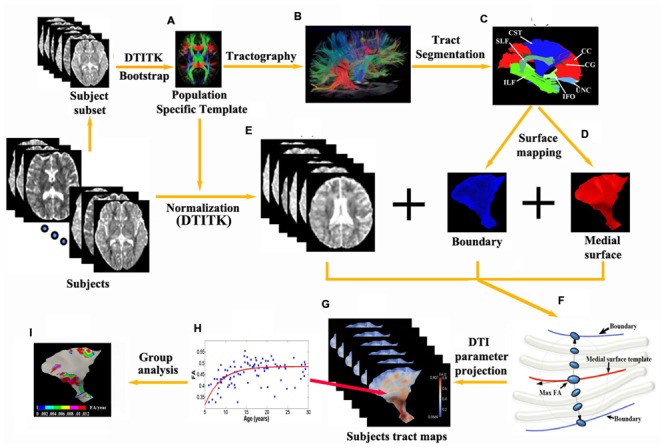
**Flowchart of the tract-specific analysis includes procedures and diagram of medial geometry. (A,E)** A deformable DTI registration algorithm, DTI-TK, is used in which image similarity is computed based on full tensor images. All subjects are aligned to a population specific template. 13 major tracts **(C)** and their corresponding medial surfaces **(D)** are manually segmented and extracted from the brain tractography of the template **(B)**. **(F)** The red curve represents the medial surface (skeleton) of the corticospinal tract. The boundaries (purple edges) are derived from the skeleton and radial field by inverse skeletonization. The vector (black arrow) lies in the tangent plane of the medial surface, and points in the direction of greatest changes in radius. DTI measurement (e.g., max FA) along the radius within the boundaries is then projected on to the individual subject’s tract map in the template space **(G)** and can be further analyzed per vertex such as exponential fits versus age over a population, etc. **(H)** yielding parametric maps [e.g., Δ(FA)/year in **I**].

Image preprocessing steps included motion and eddy current distortion corrections and diffusion tensor fitting (linear), which were performed using FSL 5.0^1^. DTI-TK^2^ was then applied to obtain a population-specific tensor template and the medial surface of major tracts from the template. **Figure 1** demonstrates a flowchart of the procedures. The achievement of the optimal spatial normalization requires a tensor template derived from subjects with equal sex distribution and similar age range. We first bootstrapped a population specific template (Zhang et al., 2007) from 12 evenly age/sex distributed subjects across the whole age range (6F/6M, 10–29 years; **Figure 1A**). In short, an initial average template was constructed as a Log-Euclidean mean of the selected DTI images to preserve WM orientation with minimal blurring (Arsigny et al., 2006). Then the template was iteratively refined by affine and non-linear registration in which image similarity is computed based on full tensor images of the subject to the template (Zhang et al., 2006, 2007). This atlas construction approach has been shown to provide a more accurate study specific template compared to the more common scalar parameter methods (Keihaninejad et al., 2013).

Using DtiStudio (Jiang et al., 2006), 13 tracts were manually segmented from the template (**Figures 1B,C**) via the fiber assignment by continuous tracking (FACT) algorithm with FA threshold 0.15 and angular threshold of 70° (Mori et al., 1999) and the skeleton of each tract was approximated by a parametric surface using the continuous medial representation framework (cm-rep, **Figure 1D**; Yushkevich et al., 2008). Boundaries and medial surfaces of 13 tracts were generated in the template including the CC (15230 vertices, 16690 mm^2^), as well as the following tracts in the left and right hemispheres: corticospinal tract (CST, left/right: 5683/8167 vertices, 4528/4131 mm^2^), cingulum (CG, left/right: 4835/5027 vertices, 600/634 mm^2^), inferior fronto-occipital fasciculus (IFO, left/right: 6615/7591 vertices, 3547/3268 mm^2^), inferior longitudinal fasciculus (ILF, left/right: 7591/8327 vertices, 2118/2270 mm^2^), superior longitudinal fasciculus (SLF, left/right: 4931/5959 vertices, 1884/961 mm^2^), and uncinate fasciculus (UNC, left/right: 6903/4931 vertices, 1270/1417 mm^2^). Note that depending on the tract characteristic, the number of vertices does not necessarily match directly with surface area (e.g., tracts with higher curvature have more vertices).

Next, all subjects were aligned to the population-specific template using DTI-TK (**Figure 1E**). Spatially normalized tensor fields were sampled for each subject along directions of radius originating from the tract skeleton to the tract boundary and the largest FA (with a minimum threshold of 0.2) of all the tensors and their corresponding MD values between the boundaries were selected for each point on the parametric skeleton for each subject (**Figure 1F**). FA and MD measurements along the surfaces were then smoothed using a surface-based diffusion smoothing kernel of approximately 8 mm FWHM (**Figure 1G**) that preserves topology of the tracts (Chung et al., 2005). FA and MD values of each vertex on each tract were mapped against age for all subjects (**Figure 1H**).

### Vertex-Based Curve Fitting of FA and MD Changes with Age

From our previous paper on a large cohort, we demonstrated that diffusion changes from childhood to adulthood followed an exponential pattern of maturation (Lebel et al., 2008). In this study, all fitting procedures described here were implemented in Matlab2014a and applied to FA and MD values of each vertex on the tract in male and female groups (recall: tractography is not performed in each individual). As some vertices may not have a significant exponential fit, linear fits were also performed at each vertex of the tracts. For linear fit, the linear equation of the form FA (or MD) = b+a^∗^age yielded *b* and *a* as the estimated y-intercept and the slope of the fitting. For exponential fitting, equations of the form FA (or MD) = C+Ae^-age/t^ yielded *C* as the estimate asymptote value, *A* as the y-intercept of the function and time constants *t* indicating the rate of development. The significance of the both linear and exponential fits were measured using *F*-test (*p* < 0.05) and corrected using random field theory at *p* = 0.01 (Worsley et al., 1999). A significant *F*-test indicates that the observed R-squared is reliable, and is not a spurious result of oddities in the data set. Thus, the *F*-test determines whether the proposed relationship between the response variable and the set of predictors is statistically reliable. Vertices with both significant linear and exponential fits were compared using Akaike Information Criterion (AIC) where a smaller AIC value indicates that one fit is better than the other (Akaike, 1974).

### Rate and Maximum Development Age of FA and MD Changes

For those vertices where exponential fits were significant and determined (as above) to be the better fit, the age in years where the maximum FA or minimum MD is reached for the individual vertices was determined at the 90% level of the asymptotes of the exponential curves from 6 to 30 years. The rate was then also calculated as the change of FA or MD per year from 6 years to the age of plateau. Only vertices with positive increasing pattern for FA (A < 0 in exponential fit) or decreasing pattern for MD (A > 0 in exponential fit) were analyzed per tract as few vertices demonstrated reversed trends (decreased FA or increased MD with age) and none of them survived multiple comparisons corrections. In addition the rate of change of FA or MD was given by the slope for the linear fits. These analyses were performed in all 13 tracts (CC and six bilateral) for males and females separately. The maximum development ages were compared between sexes per vertex to see if they overlapped, given the standard error from the exponential fits. For both FA and MD, if the development age plus/minus the standard errors per vertex overlapped between males and females, they were not considered to be significantly different. This method was used in our earlier whole tract development paper (Lebel et al., 2008).

Although the primary interest in this paper is on variable changes along the tract, mean DTI values of each tract averaged over all vertices with significant fits at the individual vertex level were determined to provide overall tract summary parameters for the male and female groups. Exponential and linear fit vertices were combined separately. To simplify this mean over the tract analysis, the FA/MD values of all the significant vertices for each tract were averaged over left and right sides (e.g., SLF.L+SLF.R).

## Results

### Mean FA and MD Changes with Age

Using the normalized, medial surface model of the tracts resulted in significant exponential/linear fitting of mean FA and MD in many regions of seven major tracts (CC, bilateral CST, SLF, UNC, ILF, IFO, CG) for both male and female groups (**Tables 1** and **2**). Overall, the percentage of vertices with significant exponential FA increases with age was 27 ± 10% (range: 15–50%) over the various tracts and was greater for exponential MD decreases over age with 51 ± 18% (range: 18–84%). Another 14 ± 8% (range: 4–36%) of the vertices for FA and 13 ± 10% (range: 1–32%) for MD showed linear changes with age per tract. Combining vertices with either significant exponential or linear fits with age, only 41 ± 9% (range: 29–56%) and 63 ± 18% (range: 26–85%) of the vertices showed changes with age for FA or MD, respectively. In general, MD showed more expansive regions that changed with age per tract. The proportion of vertices with exponential fits was greater in the females than the males for all tracts by an absolute ∼13 ± 8% for FA (range: 5–22%) and ∼12 ± 13% for MD (range: 1–40%).

**Table 1:** Percentage of vertices with significant exponential (*P_exp_*) and linear (*P_lin_*) fits and correlation (*R_exp_* and *R_lin_*), exponential time constant (*t*), absolute change of FA per year from age 6 to 30 (linear) and age (*T*) where FA reaches 90% of the plateau maximum (exponential) over those vertices with exponential fits for each tract (left + right combined) of males (M) and females (F) separately.

Tracts		Fractional Anisotropy versus Age				
	Sex	*P_exp_ (P_lin_) %*	R_exp_ (R_lin_)	*t_exp_ (SE)*	Absolute increase per year Exp (Lin)	*T_exp_(SE)* year
CST	M	22 (10)	0.59 (0.59)	5.5 (1.4)	0.005 (0.004)	18.8 (3.3)^∗^
	F	27 (4)	0.53 (0.31)	2.8 (0.6)	0.01 (0.003)	12.4 (1.4)^∗^
CG	M	28 (19)	0.56 (0.62)	5.3 (1.5)	0.008 (0.004)	18.3 (3.3)
	F	50 (10)	0.63 (0.52)	4.3 (0.9)	0.01 (0.004)	15.9 (2.1)
CC	M	19 (16)	0.61 (0.65)	5.3 (1.3)	0.006 (0.003)	18.2 (3.1)
	F	24 (8)	0.6 (0.48)	4.4 (1.0)	0.007 (0.003)	16.2 (2.2)
IFO	M	23 (16)	0.52 (0.6)	6.2 (2.0)	0.005 (0.003)	20.3 (4.7)
	F	34 (10)	0.57 (0.42)	4.1 (0.9)	0.008 (0.003)	15.4 (2.1)
SLF	M	18 (22)	0.54 (0.63)	6.8 (2.3)	0.005 (0.003)	21.6 (5.2)^∗^
	F	38 (10)	0.54 (0.47)	3.6 (0.8)	0.009 (0.003)	14.2 (1.8)^∗^
ILF	M	15 (15)	0.5 (0.56)	6.4 (2.3)	0.004 (0.003)	20.8 (5.2)
	F	21 (13)	0.45 (0.52)	5.8 (2.0)	0.004 (0.003)	19.3 (4.5)
UNC	M	20 (35)	0.5 (0.59)	7.6 (2.9)	0.003 (0.003)	23.4 (6.6)
	F	38 (9)	0.51 (0.29)	5.0 (1.4)	0.006 (0.003)	17.4 (3.2)

*CST, Corticospinal tract; CC, corpus callosum; CG, cingulum; IFO, inferior fronto-occipital fasciculus; SLF, superior longitudinal fasciculus; UNC, uncinate fasciculus; ILF, inferior longitudinal fasciculus. Tracts are listed in the order of increasing mean T averaged over both sexes for FA exponential fits. SE, standard error. ^∗^Significant sex differences.*

**Table 2:** Percentage of vertices with significant exponential (*P_exp_*) and linear (*P_lin_*) fits and correlation (*R_exp_* and *R_lin_*), exponential time constant (*t*), absolute change of MD per year from age 6 to 30 (linear) and age (*T*) where MD reaches 90% of the plateau minimum (exponential) over those vertices with exponential fits for each tract (left + right combined) of males (M) and females (F) separately.

Tracts		Mean Diffusivity versus Age				
	Sex	*P_exp_ (P_lin_) %*	*R_exp_ (R_lin_)*	*t_exp_ (SE)*	Absolute decrease per year Exp (Lin) 10^-3^mm^2^/s	*T_exp_(SE)* year
ILF	M	61 (19)	0.52 (0.47)	5.9 (1.9)	0.006 (0.004)	19.6 (4.3)^∗^
	F	72 (3)	0.39 (0.27)	2.7 (0.7)	0.014 (0.004)	12.2 (1.6)^∗^
IFO	M	53 (21)	0.57 (0.57)	7.1 (2.4)	0.005 (0.004)	22.4 (5.5)^∗^
	F	64 (4)	0.48 (0.26)	3.2 (0.8)	0.012 (0.004)	13.3 (1.8)^∗^
CC	M	35 (18)	0.55 (0.63)	6.0 (1.9)	0.007 (0.005)	19.8 (4.4)
	F	36 (5)	0.44 (0.37)	4.4 (1.3)	0.008 (0.004)	16.0 (3.1)
SLF	M	44 (41)	0.51 (0.48)	7.6 (3.0)	0.004 (0.004)	23.5 (6.8)^∗^
	F	84 (2)	0.38 (0.20)	3.3 (1.0)	0.009 (0.003)	13.7 (2.4)^∗^
UNC	M	60 (23)	0.58 (0.56)	6.9 (2.2)	0.005 (0.005)	22.1 (5.0)
	F	61 (15)	0.42 (0.28)	4.3 (1.3)	0.008 (0.005)	15.8 (3.0)
CG	M	18 (19)	0.52 (0.48)	6.7 (2.5)	0.006 (0.006)	21.4 (5.6)
	F	25 (1)	0.45 (0.33)	4.7 (1.5)	0.008 (0.004)	16.7 (3.4)
CST	M	42 (22)	0.67 (0.62)	10.3 (3.6)	0.004 (0.004)	29.6 (8.3)^∗^
	F	55 (2)	0.43 (0.31)	4.2 (1.3)	0.007 (0.003)	15.7 (2.9)^∗^

*Tracts are listed in the order of increasing mean T averaged over both sexes for MD exponential fits. ^∗^Significant sex differences.*

**FIGURE 2:**
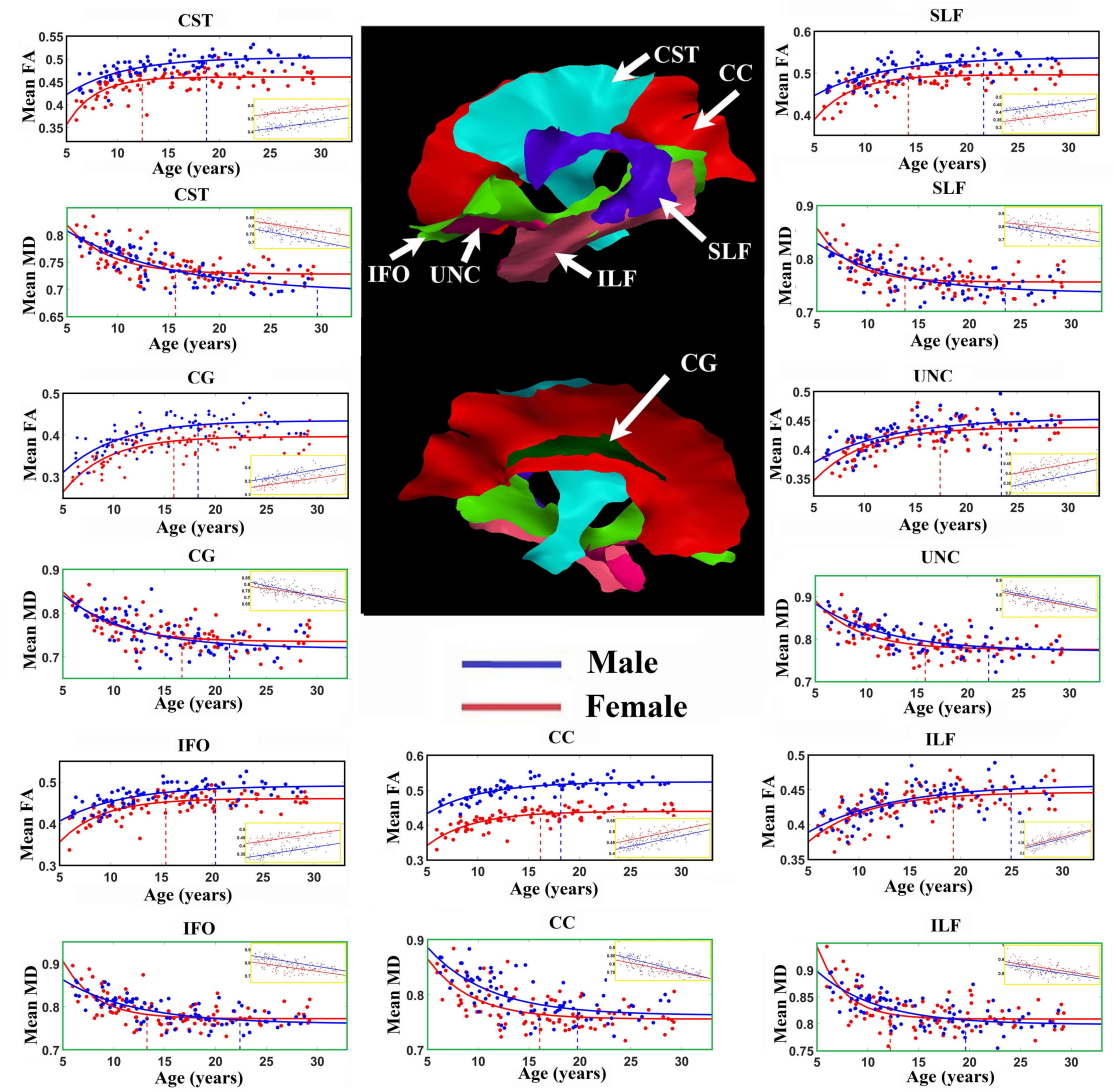
**Age-related FA increases and MD (units of 10^-3^ mm^2^/s) decreases averaged over all vertices with either exponential or linear fits (insets) of each tract in males (blue lines) and females (red lines).** Middle: Tract surfaces derived from the template are shown, but recall that only a proportion of these vertices contribute to these exponential curves and linear fit (as in **Tables 1** and **2**). Maximum FA and minimum MD plateau of each tract for both female (red) and male (blue) are marked by the vertical dotted lines. Projection and inter-hemispheric tracts such as CST and CC tend to reach the FA plateau earlier than temporal lobe association tracts such as SLF, UNC, and ILF. Generally, males have higher FA over the full age span for the exponential whereas it is quite similar for MD. There are differences in the fit parameters presented in **Tables 1** and **2**. CST, Corticospinal tract; CC, corpus callosum; CG, cingulum; IFO, inferior fronto-occipital fasciculus; ILF, inferior longitudinal fasciculus; SLF, superior longitudinal fasciculus; UNC, uncinate fasciculus.

**FIGURE 3:**
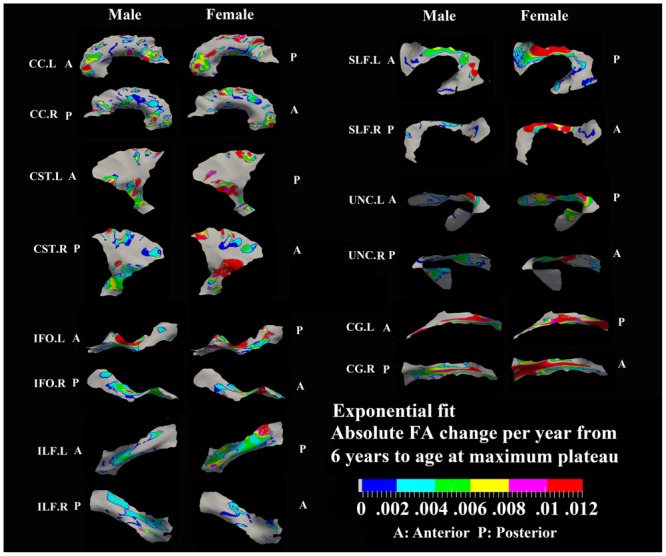
**Areas with significant exponential increase of FA and their absolute change per year from age 6 years to age where FA plateaus at its maximum value in 13 tracts.** Here, the plateau is defined as 90% of FA of the asymptote of the exponential curve from 6 to 30 years. Faster FA changes were observed in genu of CC, fronto-parietal part of SLF and inferior CST relative to the other areas of the tracts. Smaller tracts such as left ILF, IFO, and CG displayed faster changes in the posterior part of the tracts, though more pronounced in females. Gray regions are those vertices that are either better fitted with a linear model or have no significant correlation with age.

**FIGURE 4:**
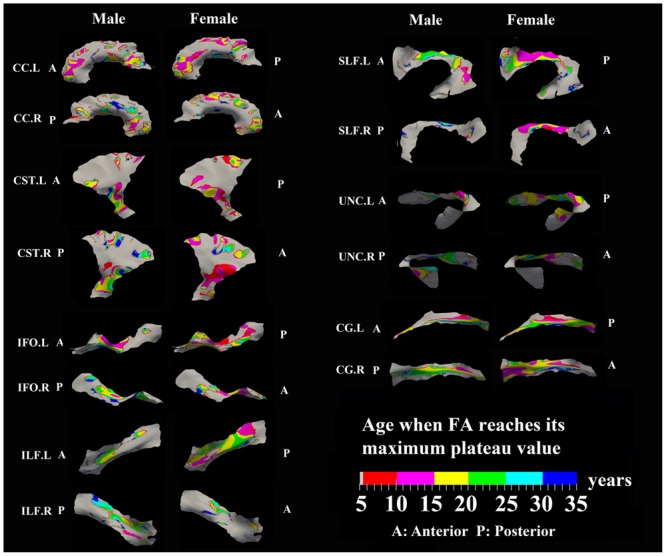
**Areas with significant exponential increase of FA and the age needed to reach the maximum plateau value (defined as 90% of maximum) in 13 tracts.** Genu of CC, inferior CST, superior portion of SLF, and posterior sections of left ILF and left IFO in females all reach their maximum level before age 15 years. Males have several regions where the maximum level is reached after 20 years, particularly in right CG, right UNC, and left SLF. Gray regions are those vertices that are either better fitted with a linear model or have no significant correlation with age.

**Figure 2** shows the average of the FA or MD over all the vertices with significant exponential changes in the combined left/right tracts, leading to several observations. The CST reaches FA maximum earliest and UNC latest; while for MD the ILF reaches MD minimum earliest and CST the latest. With the exception of these two tracts, the other five tracts each showed similar timings for their FA and MD changes with age. The FA appeared to be higher in males in most of the tracts over the full age span, whereas MD overlapped considerably between sexes with the exception of the CC. Overall, mean FA and MD of the tracts reach their plateau (T) earlier in females (12–20 years, mean of 15 years) than in males (17–30 years, mean of 21 years), as seen in **Table 1** for FA and **Table 2** for MD. Females demonstrated a significant earlier plateau of FA in CST and SLF by 6–7 years and MD in CST, SLF, IFO, and ILF by 5–15 years. The degree of absolute change per year for either FA or MD in the time up to the plateau is also typically larger in the females relative to the males. It should be noted that the vertices with significant exponential fits are not necessarily at the same locations for both males and females (shown in **Figures 3** and **4** for FA and **Figures 6** and **7** for MD).

**FIGURE 5:**
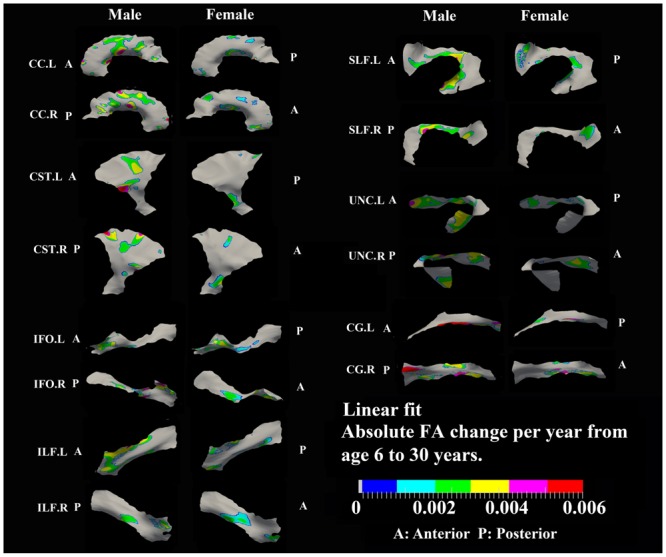
**Areas with significant linear increase of FA and their absolute change per year from age 6 years to age 30 years in 13 tracts.** Most of the linear FA increases (0.002–0.004/year) were observed in body of CC, superior bilateral CST, fronto-parietal bilateral SLF, frontal bilateral ILF, and bilateral UNC in males.

**FIGURE 6:**
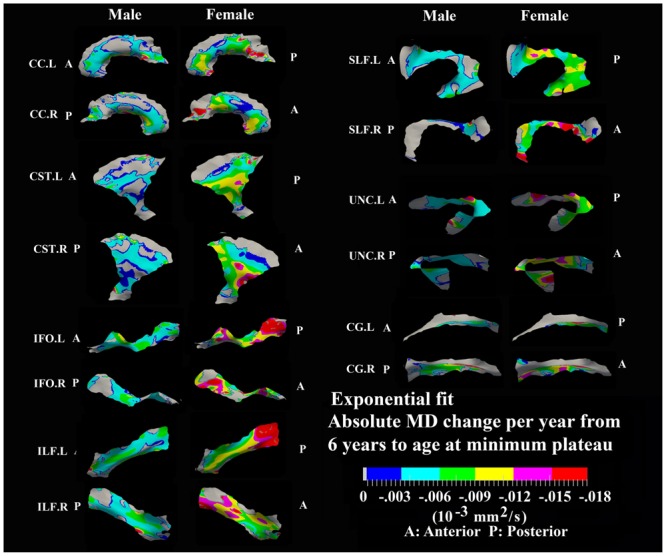
**Areas with significant exponential decrease of MD and absolute change per year from age 6 years to age where MD plateaus at its minimum value in 13 tracts.** Here, the plateau is defined as 90% of MD of the asymptote of the exponential curve from 6 to 30 years. The genu and splenium of CC have a faster decreasing rate relative to the other CC regions in both sexes. The inferior CST, fronto-parietal section of SLF, superior portion of UNC, and posterior sections of ILF and IFO in females also exhibited faster MD decreasing rate relative to other tract areas. Gray regions are those vertices that are either better fitted with a linear model or have no significant correlation with age.

**FIGURE 7:**
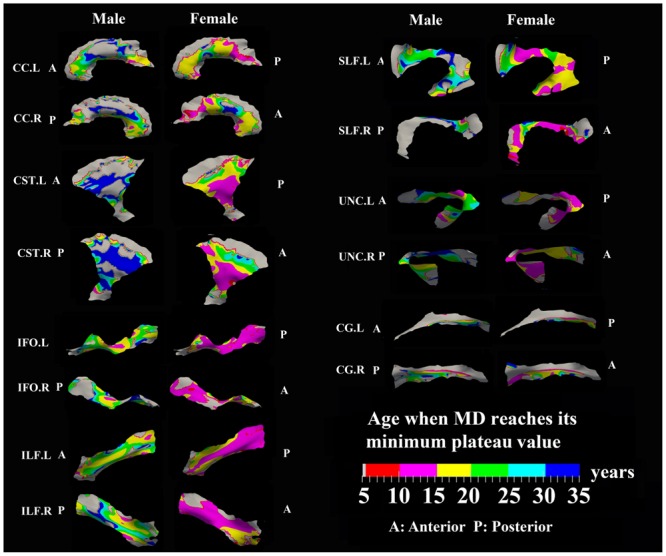
**Areas with significant exponential decrease of MD and the age needed to reach the minimum plateau value in 13 tracts.** Most tracts in females reach their minimum MD before age 15 years while males reach their minimum values after this age. Gray regions are those vertices that are either better fitted with a linear model or have no significant correlation with age.

**FIGURE 8:**
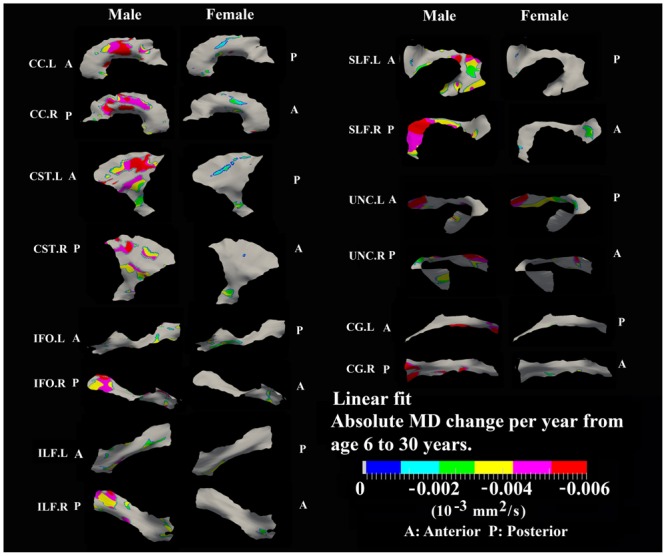
**Areas with significant linear decrease of MD and their absolute change per year from age 6 years to age 30 years in 13 tracts.** Most linear MD decreases (>0.004 10^-3^ mm^2^/s) were observed in males including body of CC, bilateral CST, posterior SLF, posterior right IFO, posterior right ILF, and frontal bilateral UNC.

### Developmental Rate and Timing of Tract-Specific FA Changes

Specific areas along each tract showed either significant exponential (**Figure 3**) or linear (**Figure 5**) patterns of increases of FA with differing rates (+FA/year). Also, the age to reach 90% of maximum FA from 6 to 30 years varied for the regions with exponential fits (**Figure 4**). Overall, similar regions of the tracts showed exponential FA increases between the male and female groups, albeit with apparent differences in rates and timing in many cases. Faster exponential FA changes were observed in genu of CC (>0.006/year for both males and females), inferior CST (0.004–0.008/year for males and >0.01 for females) and fronto-parietal part of SLF (0.004–0.006/year for males and >0.01/year for females) relative to the other areas of these three tracts. Smaller frontal tracts such as bilateral CG (>0.008/year), left ILF (>0.01/year), and left IFO (>0.01/year) in females displayed faster exponential changes in the posterior part of the tracts relative to the anterior areas. Bilateral tracts exhibited similar distribution patterns with the exception of SLF and ILF where the left side demonstrated either a more widespread pattern or faster rate. Areas of tracts with faster FA rate tended to reach their maximum value at an earlier age relative to other regions (**Figure 4**). Genu of CC and inferior CST in both sexes (before age 20 years for males and 15 years for females) and fronto-parietal part of bilateral SLF, posterior sections of left ILF and IFO in females (before age 15 years) all reach their maximum values earlier compared with other tract regions. Small regions of the right ILF in both sexes and middle sections of bilateral UNC, left SFL, right CG in males show more regions with slower development beyond 20 years to reach the maximum FA.

Most areas with linear increases of FA with age are located in the body of CC, CST, fronto-parietal SLF and UNC (0.002–0.006/year) and are mostly notable in males (**Figure 5**). The distribution of linear increases of FA in smaller tracts, IFO and ILF (0001–0.004/year), are more focused on middle to frontal areas.

### Developmental Rate and Timing of Tract-Specific MD Changes

Numerous tract vertices showed a significant exponential (**Figure 6**) or linear (**Figure 8**) pattern of decrease in MD with differences in the rate (-MD/year). As for FA, the age to reach 90% of the minimum MD from age 6 to 30 years varied across the tracts for the vertices with exponential fits (**Figure 7**). Tract-specific regions with exponential decrease of MD are much more widespread than increased FA regions in most tracts and along tract differences are much more noticeable in females, although many of the same regions as males show exponential reductions of MD. The genu and splenium of CC have a faster decreasing rate of MD (0.004–0.008 × 10^-3^ mm^2^ s^-1^/year for male and >0.008 × 10^-3^ mm^2^ s^-1^/year for females) relative to the other CC regions. Similar to the FA increasing pattern, inferior CST (>0.008 × 10^-3^ mm^2^ s^-1^/year), fronto-parietal section of SLF (>0.01 × 10^-3^ mm^2^ s^-1^/year) and posterior sections of ILF (∼0.01 × 10^-3^ mm^2^ s^-1^/year) and IFO (∼0.01 × 10^-3^ mm^2^ s^-1^/year) in females also exhibited faster MD decreasing rate relative to other tract areas. In **Figure 7**, most tracts in females reach their minimum MD before age 15 years while many areas of tracts in males displayed a prolonged maturation process after age 20 years and some well into the young adult period such as CST.

Linear decreases of MD are observed mostly in males and the spatial distribution is similar to that observed for linear FA increases in the body of CC, CST, and UNC (**Figure 8**). In addition, linear decreases of MD are greater in the posterior of SLF, right ILF and right IFO relative to other parts of those tracts in males.

### Sex Differences for FA and MD Maturation Age

**FIGURE 9:**
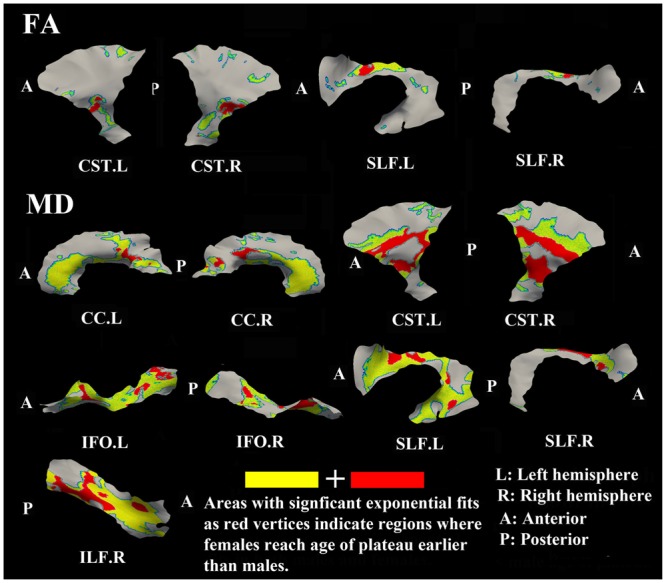
**Areas of tracts that display significant exponential fits in both males and females (yellow and red) where red indicates female age of plateau + 1 SE < male age of plateau – 1 SE (age differences between sexes that were greater than one standard error from their exponential fits are considered to be significantly different); note that the reverse pattern (males with earlier diffusion changes than females) was not observed for any vertex.** Females demonstrate earlier plateau age in the bilateral inferior CST and bilateral fronto-parietal part of SLF for FA and MD, and in the splenium of CC, bilateral IFO, and right ILF for MD. Tracts with no significant difference between the age of FA or MD plateau in males and females at the vertex level are not shown.

The surface representative tract maps of the age where FA or MD reach their plateau values (only for exponential fits of course) indicate sex differences on a per vertex basis for both FA (**Figure 4**) and MD (**Figure 7**). Sex differences were assessed by a direct vertex comparison of the ages where development is presumed to level off by comparing the overlap of age and standard error of the exponential fits for vertices that had significant exponential fits for both sexes. There were some regions (shown in red) with overlap in age ± standard error between males and females, mostly for MD (**Figure 9**). Compared with males, females reached their FA plateaus at an earlier age in inferior bilateral CST and superior bilateral SLF. Females reached the MD plateaus earlier in 8 of 13 tracts including splenium of CC, inferior bilateral CST, anterior bilateral IFO, superior bilateral SLF, and posterior right ILF.

## Discussion

Although several previous studies have used “along the tract” methodologies to investigate changes of regional diffusion parameters with development in infants (Zhu et al., 2011), young children (Johnson et al., 2013) or children/adolescents (Yeatman et al., 2012), no study has used these methods to study the maturation patterns of diffusion parameters along the major fasciculi from childhood (6 years) to adulthood (30 years). This study was an extension of our previous tractography based approach in mapping the tract maturation pattern in development where in our earlier study each tract yielded one overall mean FA or MD per subject (Lebel et al., 2008). Although informative, the whole tract averaging approaches appear to oversimplify the WM development by assuming that the diffusion parameters of the entire tract change in unison. Surface-model based TSA identified DTI variability as a function of age along the tracts. Given that diffusion parameters change in WM at unique rates with age and typically reach a developmental peak or plateau within our age window (Lebel et al., 2008, 2012), the focus here is on the vertices with exponential changes with age. The main results included: (1) confirmation of general age-related exponential FA increases and MD decreases with development from childhood to young adulthood, (2) identification of unique patterns of changes of FA or MD along major tracts in both sexes suggesting that the entire tract, as far as water diffusion is concerned, does not behave identically as a function of age, (3) demonstration of a posterior-to-anterior temporal pattern of WM maturation, and (4) demonstration of earlier maturation of FA and MD to a plateau in females relative to males in specific tract regions.

Many of the tract vertices, but not all, revealed exponential increases of anisotropy and decreases of MD with age from young childhood to adulthood in both male and female groups. These results on a subset of vertices on the surface-defined tracts (15–50% for FA and 18–84% for MD) are consistent with many studies that showed a similar pattern in typically developing children (Schneider et al., 2004; Ben Bashat et al., 2005; Zhang et al., 2005; Lebel et al., 2008; Tamnes et al., 2010; Cancelliere et al., 2013; Taki et al., 2013a), including our own with overlapping samples analyzed by individual tractography averaged over the entire tract (Lebel et al., 2008). Regions with significant exponential decreasing of MD are much broader in most tracts than those of FA increases with more pronounced differences in females. This observation is also in agreement with several previous studies indicating that decreases in MD appear to be more global in scope (Schmithorst et al., 2002; Bonekamp et al., 2007; Tamnes et al., 2010; Taki et al., 2013a). The within-tract differences of water diffusion parameters along a tract during maturation could reflect unique rates of axonal myelination and packing (Beaulieu, 2002; Song et al., 2002, 2005) that might be due to local differences in vasculature, supporting glial structure, and biochemistry throughout the brain (Ito et al., 2005; Yeh et al., 2009; Vasung et al., 2010). Light microscopic examinations of the fiber composition in the human CC also revealed complex, heterogeneous structure containing axons of different diameters and densities that vary by region (Aboitiz et al., 1992). This confirms that major WM tracts display intensive maturational changes at earlier ages that slow down and then level off at various ages depending on the tract.

When taking vertices with linear fits with age into account, the proportion of vertices showing changes with age were ∼41% on average over all tracts for FA and ∼63% for MD, supporting the notion that FA and MD are complementary measures. While some additional vertices showed linear changes of FA or MD with age, there was still a sizeable proportion of vertices in each tract that did not show any change (44–71% for FA and 15–74% for MD). One explanation would be that those regions are nearly or fully developed by the age of 6 years, thus a clear developmental trend cannot be mapped in this study. Another possible interpretation would be the methodological limitations in analyzing those surface vertices including the confounding fact of regional crossing fibers from tractography, insufficient data smoothing, inadequate spatial correspondence across subjects, strict fitting models and WM structural variability.

Relative to other portions of the same tracts, there were faster increases of FA in genu/splenium of CC, which provide inter-hemispheric connections between homologous neocortical regions, and inferior CST that serve crucial roles in sensorimotor integration. The findings of CC are consistent with previous studies that demonstrated the genu/splenium of the CC was amongst the earlier regions for FA and MD to level off with age during development (Lebel et al., 2008; Cancelliere et al., 2013). Similarly, inferior (internal capsule) and frontal (corona radiata) part of CST were also shown to display faster developing rate in a whole-brain DTI study of healthy individuals between ages 8 and 28 years (Asato et al., 2010). Furthermore, the fronto-parietal part of SLF also showed a faster developmental rate, suggesting an intensive maturation process of that section of tract from childhood to adolescence. A recent study demonstrated an early development (increased FA) of arcuate fasciculus, from 5 to 8 years that was positively correlated to the receptive/expressive language scores (Broce et al., 2015). The fronto-parietal portion of SLF is also known to be involved in early development of working memory (Nagy et al., 2004; Vestergaard et al., 2011). Association tracts such as CG, ILF, IFO, and UNC either displayed faster exponential changes in the posterior part of the tracts or did not show any age effects (gray areas) that indicate early maturity or a prolonged increase in the anterior part of the tracts. These results are in agreement with previous DTI reports of changes in diffusion parameters that are generally concentrated in more posterior regions during early stages of development (Asato et al., 2010; Colby et al., 2011).

Areas of tracts with faster FA rate tended to reach their maximum value at an earlier age relative to other regions. Genu/splenium of CC, inferior CST and fronto-parietal part of bilateral SLF all reach their peak before age 20 years (males < 20 years/females < 15 years). More importantly, posterior areas of ILF, IFO, CG, and UNC in both sexes reach their maximum values earlier (<15 years) or did not show any peak ages (<5 years) while anterior regions demonstrated longer maximum developmental timing for FA. These results are consistent with a prolonged frontal maturation pattern in the development of WM suggested in many previous neurodevelopmental studies (Flechsig, 1901; Yakovlev and Lecours, 1967; Giedd et al., 1996; Paus et al., 2001; Deoni et al., 2012; Dean et al., 2015). Recent post-mortem studies of human brain have shown that the time course of synaptogenesis is earlier in the visual and auditory cortex than in the prefrontal cortex (Huttenlocher, 1979; Huttenlocher et al., 1982; Huttenlocher and Dabholkar, 1997). Additionally, synapse elimination starts earlier in the visual cortex than in the auditory cortex, followed by the prefrontal cortex (Huttenlocher and Dabholkar, 1997). These results suggest that brain maturation starts in the occipital lobe followed by the temporal lobe and then the frontal lobe. Recent DTI development studies in both primate and human also demonstrated a similar posterior to anterior pattern suggesting prolonged myelination in frontal regions (Colby et al., 2011; Shi et al., 2013) that is consistent with the protracted trajectory of cognitive development in executive functioning domains, which similarly continues through adolescence and is known to involve processing in the frontal lobe (Luna et al., 2004, 2010). Thus, our results further demonstrate this pattern of late maturation of tracts in the frontal and temporal lobes with a tract-specific approach.

Tract-specific regions with exponential decrease of MD have delayed maturation compared with increased FA regions for most tracts (compare **Figures 3** and **4** for FA versus **Figures 6** and **7** for MD). Differences in timing of WM development has also been observed even when averaging over entire tracts in this same cohort previously (Lebel et al., 2008), and this MD delay persists into aging mostly for association tracts (Lebel et al., 2012). Another aging study using TBSS also demonstrated the timing difference of maximum development among several global diffusion measurements (FA, MD, RD) where MD also displayed a delayed maturation compared to other parameters (Westlye et al., 2010). The discrepancies between FA and MD change trajectories may reflect different FA and MD sensitivities to underlying physiologic processes or possibly less variability in the measure of MD with conventional DTI. The prevalent notion is that increases in FA are associated with organization of the tracts and their myelination and denser axon packing, whereas MD may be more sensitive to decreases of total brain water content and volume of extracellular space (Morriss et al., 1999; Beaulieu, 2002; Mukherjee et al., 2002; Hermoye et al., 2006; Dubois et al., 2008; Saksena et al., 2008; Giorgio et al., 2010). A review of many genetic dysmyelination studies in animal models has also suggested that reductions of MD may be sensitive (more so than FA) to changes in myelination (see Table 8.2 in Beaulieu, 2014), which would fit nicely with our observations of MD reductions along large portions of the tracts. However, the lack of specificity of DTI metrics to microstructure is well known and the exact interpretation of our data on diffusion changes at specific tract regions remains elusive.

Perhaps to be expected, it was shown that males have delayed maturation timing of MD decreases, and less so for FA increases, compared with females in some areas along surfaces of several large tracts. In females, the FA and MD mostly plateau before the age of 15 years, but MD has more regions than FA with protracted leveling off in the 15–25 years range. In males, MD shows later leveling off relative to FA in far more regions, mostly beyond 20 years. The findings are largely in agreement with previous voxel-based studies demonstrated that by adolescence, females have reached a developmental plateau in most diffusion parameters of the majority of tracts (e.g., CST, SLF, ILF) whereas males had a more protracted course with tracts continuing to develop into adulthood (Asato et al., 2010; Bava et al., 2011; Wang et al., 2012; Taki et al., 2013b; Simmonds et al., 2014). Sexual dimorphism in the development of WM might be attributed to different mechanisms of WM growth in males and females, specifically, an increase in axonal caliber in males and a growth in myelin content in females (Perrin et al., 2009). Increasing testosterone levels may also influence axonal caliber in males, suggesting a role for sex hormones in WM maturation (Perrin et al., 2008). It is important to point out that in our previous paper in the same cohort using mean FA and MD values over the entire tracts, significant sex differences in exponential fits were not observed (Lebel et al., 2008) suggesting an advantage for examining focal regions of tracts such as the surface-based model approach described here.

One limitation of the study is the relatively small subject number of 178 subjects. Although, there are much larger public DTI datasets available online, most of them involve multi-site scanning which raises other issues related to systematic differences of diffusion parameters. Nonetheless, a similar analysis of such data sets would be helpful for replicating our single-site findings. This study can also be compared directly to our previous tractography-based whole tract analysis in the same cohort (Lebel et al., 2008). Another caveat of the study is that we only focused on FA and MD analysis to be consistent with our previous study. However, future investigation of axial (AD) and radial diffusivity (RD) could provide more insightful information regarding brain WM development. Compared to the common spline-based along-tract strategies that assume cross-sectional symmetry along the tracts, our automated surface-model approach captures variability along more dimensions within a tract (Yushkevich et al., 2008; Zhang et al., 2010). However, a few methodological issues and limitations with this approach need to be addressed. First, DTI images with six gradient directions and deterministic tractography were used for atlas tractography that may limit the accuracy and extent of our WM tract surface construction. Although, one study found minimum differences while comparing diffusion parameters measured using six or more diffusion-encoding gradient directions with deterministic tractography (Lebel et al., 2012), in future studies, higher angular resolution diffusion images and more sophisticated tractography approaches could help overcome these limitations. However, while this would enable more accurate tracking of fuller tracts through fiber crossing regions, the effects on the diffusion tensor parameters of FA and MD would not be corrected using more directions. Second, the framework is best suited for sheet-like tracts (e.g., CC), while tube-based tracts (e.g., CG) might result in inaccurate tract surfaces due to difficulty in defining the medial surface of a tube. However, the framework can be extended to include tube-based geometrical models as developed by others (Jones et al., 2005b; Colby et al., 2012) in the future. Third, our statistical method requires the vertex-based DTI values on tract surfaces to be smoothed by a surface-based diffusion smoothing kernel (8 mm). However, surface areas/vertex densities vary among all tracts, thus, making it difficult to choose an optimal kernel size for each surface. Fourth, the number of the vertices for each tract varies depends on the size and shape of the tract surface, and is quite large that might cause oversampling of the surface with respect to the true spatial biological variation in these metrics. In future study, a vertices reduction/optimization step can be applied to overcome this issue. Fifth, we applied an atlas based fiber tract surface analysis for analyzing DTI images of participants with very different ages. The vertex-based results will depend on the accuracy of the registration of each participant with the created DTI atlas, although the use of a deformable DTI registration algorithm (DTI-TK) helps minimize errors relative to other approaches. Also by selecting the tensor with maximal FA along the spokes similar to the TBSS approach, it can lead to increased sensitivity, albeit at the cost of potentially missing significant differences in areas of adjoining fasciculi (Yushkevich et al., 2008). Lastly, although informative, cross-sectional studies are limited because they cannot provide information about change within individuals. However, future longitudinal tract specific study of brain development using this surface-model approach can be performed to demonstrate within-subject maturation along major tracts from childhood to adulthood.

## Conclusion

In this study, diffusion anisotropy increased and MD decreased with age in distinct regions of WM tracts with greater changes in childhood that level off into adolescence and even early adulthood depending on the tract. TSA along the major tract surfaces uncovered unique patterns of localized rate and plateau timing of FA and MD changes with age, suggesting differences in developmental maturation. Notably, many tract regions showed an earlier leveling off for FA and MD in females than in males, highlighting sex differences in WM development not appreciated by prior “whole-tract” analyses. The proposed methodology that focuses on specific tracts can provide regional diffusion measures along tracts that will provide insight on brain development and disorders.

## Author Contributions

ZC and CB designed/analyzed the data and wrote the manuscript; HZ and PY helped designing data processing procedures; ML helped analyzing the data.

## Conflict of Interest Statement

The authors declare that the research was conducted in the absence of any commercial or financial relationships that could be construed as a potential conflict of interest.

## Footnotes

1 http://fsl.fmrib.ox.ac.uk/fsl/fslwiki

2 http://dti-tk.sourceforge.net/
